# A Comparison of Somatic Variables of Elite Ice Hockey Players from the Czech ELH and Russian KHL

**DOI:** 10.1515/hukin-2015-0019

**Published:** 2015-04-07

**Authors:** Petr Kutáč, Martin Sigmund

**Affiliations:** 1 Human Motion Diagnostics Center, University of Ostrava, Ostrava, Czech Republic.; 2 Faculty of physical culture, Palacký University, Olomouc, Czech Republic.

**Keywords:** ice hockey, morphological variables, body composition, segmental analysis

## Abstract

The goals of this study were to evaluate the basic morphological variables of contemporary elite ice hockey players, compare the parameters of players in the top Russian ice hockey league (KHL) with those of the top Czech ice hockey league (ELH), and to evaluate the parameters of players according to their position in the game. The research participants included 30 KHL players (mean age: 27.1 ± 5.1 years) and 25 ELH players (mean age: 26.4 ± 5.8 years). We determined body height, body mass, and body composition (body fat, fat-free mass, segmental fat analysis). All measurements were performed at the end of preseason training. The KHL players had the following anthropometric characteristics: body height 182.97 ± 5.61 cm (forward) and 185.72 ± 3.57 cm (defenseman), body mass 89.70 ± 5.28 kg (forward) and 92.52 ± 4.01 kg (defenseman), body fat 10.76 ± 0.63 kg (forward) and 11.10 ± 0.48 kg (defenseman), fat-free mass 78.94 ± 4.65 kg (forward) and 81.42 ± 3.52 kg (defenseman). The values for ELH players were as follows: body height 182.06 ± 5.93 cm (forward) and 185.88 ± 7.13 cm (defenseman), body mass 88.47 ± 7.06 kg (forward) and 89.36 ± 10.91 kg (defenseman), body fat 12.57 ± 2.89 kg (forward) and 11.91 ± 3.10 kg (defenseman), fat-free mass 75.93 ± 6.54 kg (forward) and 77.46 ± 7.89 kg (defenseman). The results indicate that it is beneficial to ice hockey players to have increased body mass and lower body fat, which leads to higher muscle mass, thus enabling a player to perform at the highest level and meet the specific challenges of the game.

## Introduction

To provide effective training for a sport, it is important to understand the structure of performance and the level of relevant factors that are a part of that structure. There are many investigators trying to determine the broad structure of performance taking into consideration all factors that affect sport results. Therefore, models of the structure of performance have been created both at the general level (Grossern and Zintl, 1999; Schnabel et al., 2008) and for specific sports, including team sport games.

These models include somatic variables, in addition to many other factors. The somatic factors, sometimes called morphological or constitutional, most frequently include body height, body mass, and body composition.

From the physiological perspective, ice hockey is an intermittent physical activity (Montgomery, 2000). Experts on ice hockey and similar sports focus on the composition and quantification of the individual elements of competition loads (Hoff et al., 2005; MacLean, 2008; Manners, 2004; Montgomery, 2006; Quinney et al., 2008; Vescovi et al., 2006). At present, training of elite ice hockey players mainly focuses on muscle strength, aerobic capacity, anaerobic power, speed, and agility (Behm et al., 2005; Burr et al., 2008; Hoff et al., 2005; MacLean, 2008; Manners, 2004; Montgomery, 2006; Quinney et al., 2008; Vescovi et al., 2006). The results of regular excercise are reflected in individual morphological parameters and the morphological phenotype of the athlete (Blanchard, 1995). It is obvious that excellent motor skills and a high level of physical conditioning are needed to achieve the highest level of performance. For ice hockey players, the somatic variables that are important for performing the game at the highest level must also be optimized (Barzilay, 2002; Green et al., 2006; Nadeau et al., 2008; Quinney et al., 2008).

Morphological variables have become as important for ice hockey as for other sports as shown by the incorporation of somatic parameters together with excercise testing into the functional diagnostics protocol for ice hockey players (Quinney et al., 2008; Roczniok et al., 2013). The morphological characteristics of ice hockey players have changed, not only as a result of the secular trend, but also because of sports selection that started in 1970. Between 1928 and 2010, body height and mass of Czech players increased by a mean of 10.9 cm and 18.9 kg, respectively, with marked increases occurring in the 1970s and 1980s. Between 1998 and 2010, there was another marked increase in the mean values of body height and mass of 2.1–3.2 cm and 3.6–7.9 kg, respectively (Sigmund et al., 2012). However, the effects of these changes in somatic variables on players’ performance and function in sports games are unclear.

We can assess the sports performance of an ice hockey player based on the league in which he plays (e.g. first league, second league, etc.) and by his participation in national competitions (Ice hockey Extraleague (ELH), Russian continental league (KHL), Canadian-American National Hockey League (NHL) and others). The aim of our study was to compare the somatic variables of players in the Russian KHL and those of the top Czech ice hockey league, the ELH, who regularly participate in playoff games. The variables of the KHL players can be considered representative of ice hockey players, since the KHL is regarded by professionals to be the second-best ice hockey league in the world, after the Canadian-American National Hockey League. Therefore, we believe that our findings will allow us to assess the effect of somatic variables on sports performance of professional ice hockey players.

In addition to the goaltender, there are 2 positions in ice hockey, the forward and defenseman. A secondary aim of this study was to compare the variables of the players based on their position in the game to determine if it affects somatic characteristics. This information may aid coaches in the selection of players with characteristics appropriate for a particular position and also for the development of training protocols specific to the position.

The aim of this study was to evaluate basic morphological variables of contemporary elite ice hockey players and to compare the variables of KHL players with those of ELH players. The secondary aim was to compare the monitored variables with regard to the game position of the players.

## Material and Methods

### Participants

The study included 30 KHL players (19 forwards and 11 defenseman) with a mean age of 27.1 ± 5.1 years and 25 ELH players (16 forwards and 9 defenseman) with a mean age of 26.4 ± 5.8 years. All subjects had professional contracts. Both the KHL and ELH teams consisted of regular play-off participants.

### Procedures

The chosen somatic variables were assessed after preseason training, before the start of the autumn season. Body height (BH) was measured using an A-213 Anthropometer (Trystom, Czech Republik). Body mass (BM) and body composition were evaluated with the use of a tetrapolar bioimpednace scale Tanita BC 418 MA (Tanita corporation, Japan). This mono frequency analyzer uses the method of bioelectric impedance measuring at frequency of 50 kHz. The measurements were carried out in the Athletic mode. Total body fat (BF), segmental analysis of BF and fat free mass (FFM) were evaluated. On the basis of the values of body height and mass, the body mass index $\left(\text{BMI}=\frac{\text{FM}\;(\text{kg})}{{\text{FH}}^{2}\;(\text{m})}\right)$ was calculated. For the evaluation of fat free mass in relation to body height the fat-free mass index $\left(\text{FFMI}=\frac{\text{FFM}\;(\text{kg})}{{\text{FH}}^{2}\;(\text{m})}\right)$ was calculated.

### Statistical analysis

Data normality for sets with a sufficient number of subjects (n > 10) was verified using the Shapiro-Wilk test. The independent t-test was used to assess differences in mean values for statistical significance (forwards). The statistical significance of differences in the mean values of data sets with a low number of subjects (n < 10) was assessed using the nonparametric Mann-Whitney test (defenseman). The level of statistical significance for all tests was set at p = 0.05. Practical significance was also determined for values that were statistically significant and it was determined by calculating effect size (ES) using Cohen’s *d* guidelines. The *d* value at the level of 0.2 means a minor change, 0.5 an intermediate change and 0.8 a major change (Cohen, 1988). The anthropometric variables of the ELH players in our study were assessed with regard to the 2010 normative values of ELH players (Sigmund et al., 2012) using the normalization index (N_i_): $N_{i}=\frac{M_{1}-M_{2}}{SD_{2}}$, M_1_ = mean value of the our ELH players, M_2_ = mean value of the ELH players (Sigmund et al., 2012), SD_2_ = standard deviation of the ELH players (Sigmund et al., 2012).

The N_i_ value in the range of ±0.75 SD shows an average development of the indicator, in the range from ±0.75 to 1.5 SD a below average (above average) development of the indicator and the value above ±1.5 SD means a highly below average (above average) development. Data were analysed using PASW Statistics ver 19.0 software (IBM Company, USA).

The study protocol was approved by the Ethics and Research Committee of the Ostrava University. All participants signed an informed consent form.

## Results

The Results section presents the analysis of the selected somatic variables, a comparison of the values of the KHL and ELH players, and the comparison of values of the forwards and defensemen in monitored competitions.

This section also includes a comparison of the values drawn from the anthropometric parameters in the study of ELH players with previously reported values of other ELH players. The resulting N_i_ value of the ELH forwards (BH = 183.30 ± 5.60 cm; N_i_ = −0.21SD and BW = 86.60 ± 7.30 kg; N_i_ = 0.25SD) and ELH defensemen (BH = 185.00 ± 5.20 cm; N_i_ = 0.16SD and BW = 89.50 ± 7.30 kg; N_i_ = −0.01SD) shows a mean value of body height and mass compared to other EHL players. Since Sigmund et al. (2012) studied all elite team players, we can consider the body mass and height of our study subjects to be representative of Czech ELH players.

The mean values of the somatic variables, including segmental body fat values, of the forwards and defensemen are shown in Tables 1 and 2, respectively.

When considering all the results, we found statistically significant lower BF (kg) and higher FFM (kg) in KHL players. The practical significance of those variables was also confirmed: it was high for BF (kg) and intermediate for FFM (kg). As for the other parameters, no statistically significant differences were observed.

Segmental analysis of the ELH and KHL forwards revealed that fat was evenly distributed on the right and left extremities, with slightly more fat on lower extremities. There were no statistically or practically significant differences in fat distribution among individual segments, although the ELH forwards had more fat on their trunks (2.09 %).

The BM, BMI, FFMI, and FFM of the ELH defensemen were significantly lower than the values for the KHL defensemen. The practical significance of the BMI and FFMI of the defensemen was high, intermediate for FFM and low for BM. The significantly higher BMI value in KHL defensemen was a result of the same body height but higher body mass. The higher body mass without any differences in the body fat representation also leads to higher FFM and FFMI values in KHL defensemen.

Segmental analysis of the ELH and KHL defensemen found that fat was evenly distributed on the right and left extremities, with slightly more fat on lower extremities. The ELH players had lower BF (%) than the KHL players in all segments except for the trunk, but the differences were not significant.

The differences between the forwards and defensemen of the Czech ELH and Russian KHL were not statistically significant. Therefore, we did not assess the practical significance. The differences found in BF (kg %) and FFM (kg) ranged toward the measurement error (Heyward and Wagner, 2004). Only the differences in BH and BM were more significant. In both leagues, the forwards were shorter and lighter than the defensemen.

## Discussion

The significance of somatic variables for the performance of ice-hockey players is indicated by the inclusion of these variables into the structure of sports performance in this game (Nadeu et al., 2008) and also their inclusion into comprehensive player assessment in the NHL initial draft. The development of somatic variables together with strength of the upper and lower extremities, as well as aerobic and anaerobic capacity also predict possible performance in youth players in the NHL (Tarter et al., 2009). If the overall index of the above mentioned variables in youth forwards and defenders is around 90 percentiles in a draft, the probability of becoming a member of an NHL team within 4 years is around 60 – 72% (Tarter et al., 2009). The basic somatic variables (body height and mass) are also used as characteristics of world class players included in the ranking of the International Ice Hockey Federation (IIHF). An analysis of these data suggests a trend of an increasing level of development of the aforementioned variables along with an increasing performance level (Sigmund et al., 2014). The mentioned values indicate that in today’s elite players the average value of body height is 184.3±5.79 cm. The values of body height fully correspond with the values given for NHL players ranging from 182 to 187 cm (Montgomery, 2006; Quinney et al., 2008). The mentioned average values of body height also corresponded with average values of our monitored ELH and KHL players. A detailed comparison revealed however that the specified height range was met by only 12 KHL players (40%) and 7 ELH players (28%). With respect to individual ice-hockey positions, an analysis of body height of the best players indicates that the highest values of body height combined with the highest performance are achieved by goalkeepers followed by defenders and forwards. This fact was obvious when comparing the body height of our monitored defenders and forwards (although these differences were not statistically significant).

An analysis of body weight in today’s elite players in the IIHF ranking indicates an average value of body mass of 88.1 ± 7.37 kg (Sigmund et al., 2014). However, in the most prestigious world league, the NHL requirements for basic somatic variables are significantly higher. This particularly includes the values of body mass which is between 91 and 94 kg (Burr et al., 2008; Montgomery, 2006; Quinney et al., 2008). The highest values of body mass are specified for ice-hockey defenders, lower for forwards and the lowest for goalkeepers. This also corresponds with the differences between forwards and defenders in our monitored KHL players, not in ELH players. The range of body mass values in NHL players was met by 12 KHL players (40%) and 4 EHL players (16%). It should be noted that especially with regard to body mas the players of the Czech ELH are behind current world trends. According to the level of basic somatic variables and with respect to ice-hockey positions and possible relationships in the context of sports performance, six types of ice-hockey forwards are differentiated, i.e. a Playmaker, a Sniper, a Two-Way Forward, a Power Forward, a Grinder and an Enforcer. The highest requirements for the development of body height and mass are placed on the Power Forward, Grinder and Enforcer. Common features of these types of forwards are the use of body size in a game, development of strength abilities and general game aggression. As far as ice-hockey defenders are concerned, there are four types of players: an Offensive D-Man, a Defensive D-Man, a Two-Way D-Man and an Enforcer D-Man. The use of highly developed somatic characteristics is particularly significant for the Defensive and Enforcer types. These defenders take advantage of their physical qualities in their active approach to the opponents with highly developed strength abilities, highly aggressive play and overall durability. The above mentioned ice-hockey positions and selected player types indicate the significance of optimum development of somatic variables as a significant predictor of success.

The proportionality of the monitored players is indicated by body mass index (BMI) values. In players at a top performance level the BMI value ranges between 25.5 and 27.15 kg/m2 (Hoff et al., 2001; Montgomery, 2006; Quinney et al., 2008). This range was met by 16 KHL players (53%) and 9 EHL players (36%). Although the mentioned values indicate pre-obesity, obviously the increased weight values are caused by a higher proportion of fat-free mass (FFM), i.e. also muscles. This also corresponds with the increased FFMI values that we observed in the present study. In common population the value of this index is between 15.1 and 19.9 kg/m2 (Bahadori et al., 2006; Kyle et al., 2003). Increased FFMI values correspond with a lower proportion of fat mass. In elite players the proportion of fat mass is between 8 and 12.5% depending on the measurement methods applied (Agre et al., 1988; Green et al., 2006; Montgomery, 2006; Papapanagiotou et al., 2011; Sigmund and Dostálová, 2011). A direct effect of fat mass was revealed in skating speed measurement. Pottiger et al. (2010) claim a negative correlation between skating speeds and fat mass. To assess more complex game performance, the systems of +/− points or net scoring chances in a match throughout a season are used. These parameters are not directly influenced by the proportion of fat mass provided that the volume of fat mass of a player is in the optimum range (Green et al., 2006; Peyer et al., 2011). Thus, fat mass is not a predictor of performance. On the contrary, the development of aerobic capacity and strength development indicators in selected muscle groups are significantly correlated with overall player success (Peyer et al., 2011). Fat mass higher than 12.5% was observed in 17 KHL players (68%) and 14 EHL players (46%). The mentioned analysis indicates that players in the Russian KHL have a higher level of development of the monitored variables. Lower fat mass in these players, which is associated with higher body mass, leads to increased FFM, which increases the potential for strength development. In both ice-hockey positions the difference is significant at a medium level of significance. In relation to the typology of ice-hockey defenders and forwards the observed differences can be considered a significant factor influencing player’s performance.

Not every physical characteristic could be expected to play a role in this selection process, but two that are important and for which substantial data exist, are body height and mass (Norton and Olds, 2001). Physical fitness measures and anthropometric data are valuable in helping predict hockey playing potential. With respect to the playing position it is possible to state that anthropometry should be used when comparing elite forwards, whereas peak anaerobic power and the fatigue rate are more useful for differentiating between defensive players (Burr et al., 2008).

## Conclusions

We concluded that the morphological variables we assessed in ice hockey players from the top Czech and Russian leagues were more favourable for the KHL players. There were significant differences in body mass and fat free mass. The results of this study indicate that increased body mass with a decreased body fat ratio is advantageous for ice hockey players, due to the increased muscle mass (represented by FFM and FFMI). The increased muscle mass ratio enables the player to perform better and meet the specific challenges of the game. With regard to morphological characteristics that we assessed and their transfer into the area of sports performance, the Russian elite players had better variables than the Czech elite players. Both the Czech and Russian defensemen had parameters indicating greater robustness.

## Figures and Tables

**Table 1:** Mean Variables of the Forwards and Descriptive Statistics

Variables	ELH (n=16)	KHL (n=19)	DIFF	*d*

	M±SD	M±SD		
BM (kg)	88.47±7.06	89.70±5.28	−1.23^ns^	-
BH (cm)	182.06±5.93	182.97±5.61	−0.91^ns^	-
BMI (kg/m^2^)	26.69±1.88	26.99±1.92	−0.29^ns^	-
FFMI (kg/m^2^)	22.88±1.25	23.63±1.58	−0.72^ns^	-
BF (kg)	12.57±2.89	10.76±0.63	+1.81^*^	0.90
BF (%)	14.18±3.06	12.51±3.10	+1.66^ns^	-
FFM (kg)	75.93±6.54	78.94±4.65	−3.00^*^	0.53
Segmental Analysis				
RL (BF %)	14.01±2.89	14.56±2.73	−0.54^ns^	-
LL (BF %)	14.37±2.47	14.98±2.60	−0.60^ns^	-
RA (BF %)	11.92±1.93	11.73±2.37	+0.19^ns^	-
LA (BF %)	12.03±2.16	10.97±2.18	+1.05^ns^	-
Trunk (BF %)	14.71±4.03	12.62±4.33	+2.09^ns^	-

ELH – Czech ice hockey extra-league, KHL – Russian continental league, n – frequency, BM – body mass, BH – body height, BMI – body mass index, FFMI – fat-free mass index, BF – body fat, FFM – fat free mass, RL – right leg, LL – left leg, RA – right arm, LA – left arm, M – mean, SD – standard deviation, DIFF – difference, d – effect size,

* p<0.05,

^ns^ not significant

**Table 2:** Mean Variables of the Defensemen and Descriptive Statistics

Variables	ELH (n=9)	KHL (n=11)	DIFF	*d*

	M± SD	M± SD		
BM (kg)	89.36±10.91	92.52±4.01	−3.16^*^	0.40
BH (cm)	185.88±7.13	185.72±3.57	+0.16^ns^	-
BMI (kg/m^2^)	25.76±1.44	26.83±1.08	−1.07^*^	0.85
FFMI (kg/m^2^)	22.36±0.94	23.66±0.95	−1.24^*^	1.31
BF (kg)	11.91±3.10	11.10±0.48	+0.81^ns^	-
BF (%)	13.13±2.02	11.88±2.49	+1.25^ns^	-
FFM (kg)	77.46±7.89	81.42±3.52	−3.96^*^	0.67
Segmental Analysis				
RL (BF %)	13.60±1.81	14.55±1.61	−0.95^ns^	-
LL (BF %)	13.40±1.81	14.80±1.57	−1.4^ns^	-
RA (BF %)	10.52±2.23	12.05±3.23	−1.53^ns^	-
LA (BF %)	10.13±2.45	11.01±3.08	−0.88^ns^	-
Trunk (BF%)	13.56±2.48	11.93±3.11	+1.63^ns^	-

ELH – Czech ice hockey extra-league, KHL – Russian continental league, n – frequency, BM – body mass, BH – body height, BMI – body mass index, FFMI – fat free mass index, BF – body fat, FFM – fat free mass, RL – right leg, LL – left leg, RA – right arm, LA – left arm, M – mean, SD – standard deviation, Diff – difference, d – effect size,

* p<0.05,

^ns^ not significant

**Table 3:** Differences in the Variables between Forwards and Defensemen of the Czech ELH and Russian KHL

Variables	ELH forwards - defensemen	KHL forwards - defensemen
BM (kg)	−0.89^ns^	−2.82^ns^
BH (cm)	−3.82^ns^	−2.73^ns^
BMI (kg/m^2^)	+0.93^ns^	+0.16^ns^
FFMI (kg/m^2^)	+0.52^ns^	0.00^ns^
BF (kg)	+0.66^ns^	−0.33^ns^
BF (%)	+1.05^ns^	+0.63^ns^
FFM (kg)	−1.53^ns^	−2.48^ns^

*Difference values in* *Table 3** were calculated from the mean values presented in **Tables 1** and **2*

ELH – Czech ice hockey extra-league, KHL – Russian continental league, BM – body mass, BH – body height, BMI – body mass index, FFMI – fat free mass index, BF – body fat, FFM – fat free mass,

^ns^ not significant
